# Water-Induced Nanometer-Thin Crystalline Indium-Praseodymium Oxide Channel Layers for Thin-Film Transistors

**DOI:** 10.3390/nano12162880

**Published:** 2022-08-22

**Authors:** Wangying Xu, Chuyu Xu, Zhibo Zhang, Weicheng Huang, Qiubao Lin, Shuangmu Zhuo, Fang Xu, Xinke Liu, Deliang Zhu, Chun Zhao

**Affiliations:** 1 Department of Physics, School of Science, Jimei University, Xiamen 361021, China; 2 College of Materials Science and Engineering, Shenzhen University, Shenzhen 518000, China; 3 Shenzhen Key Laboratory of Ultraintense Laser and Advanced Material Technology, Center for Advanced Material Diagnostic Technology, and College of Engineering Physics, Shenzhen Technology University, Shenzhen 518118, China; 4 Department of Electrical and Electronic Engineering, Xi’an Jiaotong-Liverpool University, Suzhou 215123, China

**Keywords:** water-induced, indium-praseodymium oxide, nanometer-thick, oxide thin-film transistors

## Abstract

We report water-induced nanometer-thin crystalline indium praseodymium oxide (In-Pr-O) thin-film transistors (TFTs) for the first time. This aqueous route enables the formation of dense ultrathin (~6 nm) In-Pr-O thin films with near-atomic smoothness (~0.2 nm). The role of Pr doping is investigated by a battery of experimental techniques. It is revealed that as the Pr doping ratio increases from 0 to 10%, the oxygen vacancy-related defects could be greatly suppressed, leading to the improvement of TFT device characteristics and durability. The optimized In-Pr-O TFT demonstrates state-of-the-art electrical performance with mobility of 17.03 ± 1.19 cm^2^/Vs and on/off current ratio of ~10^6^ based on Si/SiO_2_ substrate. This achievement is due to the low electronegativity and standard electrode potential of Pr, the high bond strength of Pr-O, same bixbyite structure of Pr_2_O_3_ and In_2_O_3_, and In-Pr-O channel’s nanometer-thin and ultrasmooth nature. Therefore, the designed In-Pr-O channel holds great promise for next-generation transistors.

## 1. Introduction

Metal oxide thin-film transistors (TFTs) are gradually becoming a key technology for applications in flat-panel displays including AMLCD and AMOLED [1,2,3,4,5,6,7,8,9]. Among a variety of metal oxides, indium oxide (In_2_O_3_) stands out due to its extraordinarily high field effect mobility (20–50 cm^2^/Vs) and optical transparency [10,11,12]. Moreover, previous studies indicate that In_2_O_3_ thin film could be grown from a novel aqueous solution route, providing great potential for cost-effective and high throughput green manufacturing [13,14]. Despite these remarkable assets, however, pristine In_2_O_3_ TFTs still suffer from high leakage current, large negative threshold voltage, as well as poor operational stability [1]. It is revealed that the intrinsic drawbacks of In_2_O_3_ channel are attributed to the oxygen vacancy-related defects. Consequently, metal cation is introduced to suppress oxygen vacancy defects and yield ternary oxide with enhanced electrical performance [15,16,17,18].

Previous investigations suggested that low electronegativity, a low standard electrode potential, and a strong dopant–oxygen bond strength are necessary for a viable dopant [19]. Among the potential doping candidates, praseodymium (Pr) holds a low standard electrode potential (−2.353 V), low electronegativity (1.1), as well as high Pr-O bonding (740 kJ/mol) [15]. Besides, the substitution of In with Pr will not bring in additional electrons since they share the same valence [16,17]. Pr_2_O_3_ and In_2_O_3_ hold the same bixbyite structure, meaning the incorporation of Pr will maintain low defect states [16]. Consequently, Pr is considered to be a superior candidate to suppress oxygen vacancy-related defects and enhance the electrical performance of pristine In_2_O_3_ TFTs. However, In-Pr-O TFTs has not been reported yet.

Here, we demonstrate, using aqueous solution processing, the synthesis of ultrathin (~6 nm) near-atomic smoothness (RMS: ~0.2 nm) crystalline Pr-doped In_2_O_3_ (In-Pr-O) layers for the first time. The aqueous precursors are insensitive to ambient moisture and hence are easy to handle. In addition, the Pr doping ratio can be tuned simply by changing the ratios between In and Pr precursors. The microstructural, chemical, and electrical analyses confirm that the incorporation of Pr could effectively suppress the oxygen vacancy-related defects, hence enhancing the performance and bias stress stability of the device. The optimized In-Pr-O TFT demonstrates state-of-the-art electrical performance.

## 2. Experimental Section

**Figure 1:**
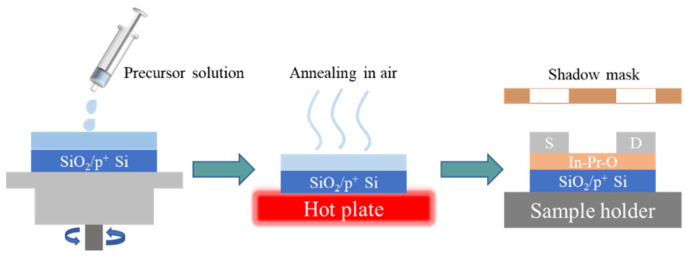
Schematic showing the fabricated In-Pr-O TFTs.

The 0.2 M In-Pr-O precursor solutions for spin-casting were prepared by mixing InN_3_O_9_∙xH_2_O and PrN_3_O_9_∙xH_2_O in DI water with Pr ratios of 0, 2, 5, and 10 mol%. The as-prepared In-Pr-O solutions were stirred rigorously at room temperature and then filtered before spin-casting. The In-Pr-O layers (~6 nm) were deposited by spin coating the precursor solutions on pre-cleaned SiO_2_ (100 nm)/p^+^-Si substrates and pre-baked at 150 ℃ and then annealed at 350 ℃ for 1 h. Finally, Al source and drain electrodes (100 nm-thick) were deposited and patterned by shadow masks with a width of 1500 μm and a length of 100 μm to finish manufacturing the In-Pr-O devices. A simple schematic diagram of the In-Pr-O TFTs preparation process is shown in Figure 1.

The crystallinities of the In-Pr-O layers were revealed by grazing incident X-ray diffraction. By using X-ray reflectivity, the thicknesses of the In-Pr-O thin films were verified. The morphologies of the In-Pr-O thin films were observed by Atomic force microscopy. X-ray photoelectron spectroscopy analysis was performed to determine the chemical properties of In-Pr-O thin films. Ultraviolet-visible and photoluminescence spectra of In-Pr-O thin films were also recorded. The typical device performances of In-Pr-O TFTs were measured by a semiconductor parameter in ambient environment at room temperature.

## 3. Results and Discussion

**Figure 2:**
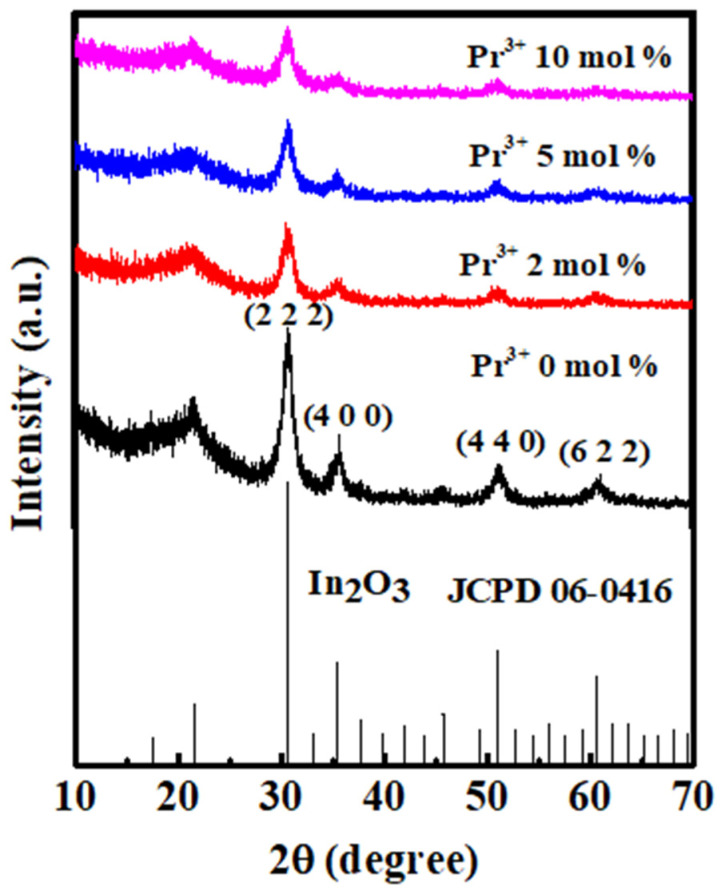
Grazing incidence X-ray diffraction patterns for In-Pr-O layers with 0, 2, 5, and 10 mol% Pr concentration.

The grazing incidence X-ray diffraction data of In-Pr-O layers with indicated Pr ratios are displayed in Figure 2. X-ray diffraction analysis reveals that the aqueous solution-processed In-Pr-O films are polycrystalline with (222) the dominant peak as well as weaker (400), (440), and (622) reflections, all belonging to the pristine In_2_O_3_ lattice [11]. Therefore, the incorporation of Pr does not break the In_2_O_3_ structure. The lattice constants of the In-Pr-O films could be extracted from the prominent peak positions of (222). Hence, the lattice constants of 0, 2, 5, and 10 mol% Pr-doped In_2_O_3_ are calculated to be 10.098 ± 0.003, 10.111 ± 0.009, 10.124 ± 0.005, and 10.129 ± 0.002 Å, respectively [10]. Since the Pr^3+^ has a lager ionic radius (0.1013 Å) than In^3+^ (0.0800 Å), the substitution of Pr^3+^ for In^3+^ will expand the lattice and leads to the increase of the lattice constant. Similar phenomena also appeared in previous research [16,19,20]. Furthermore, the (222) peak intensity becomes weaker after Pr incorporation, suggesting the decrease of crystallinity.

**Figure 3:**
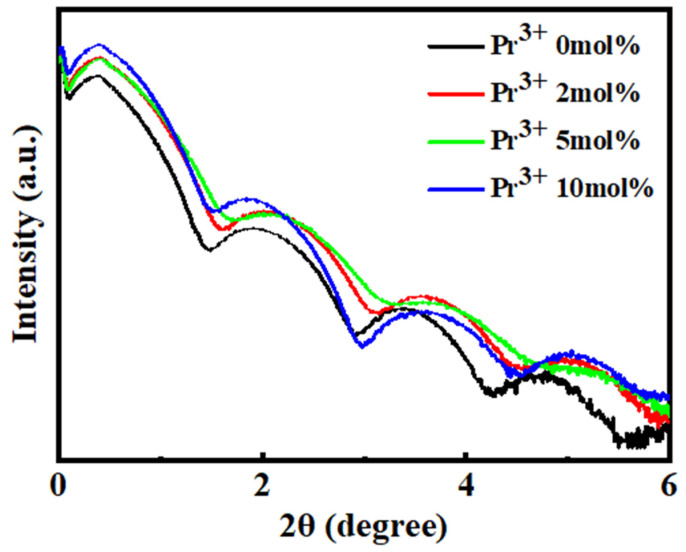
X-ray reflectivity patterns of In-Pr-O films with various Pr contents.

To evaluate the thicknesses of the In-Pr-O thin films, we performed X-ray reflectivity characterization, as shown in Figure 3. The observed thickness of the In-Pr-O films with 0, 2, 5, and 10% Pr contents were 5.74 ± 0.24, 5.78 ± 0.21, 5.83 ± 0.17, and 6.15 ± 0.19 nm, respectively. The thickness of the In-Pr-O film gradually increases with the increase of Pr doping, but it is well maintained at around 6 nm. The slight increase of thickness may be due to the relatively higher viscosity of Pr precursor than that of In.

**Figure 4:**
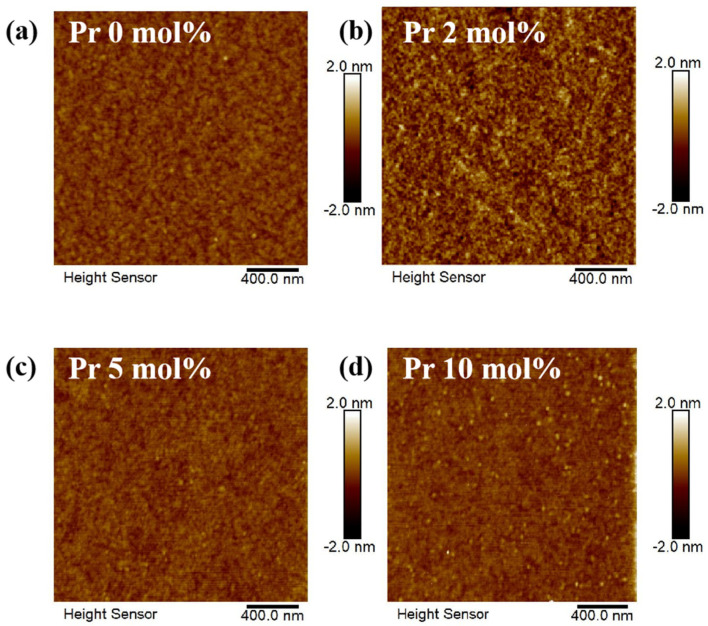
Images of In-Pr-O films with with Pr ratios of (**a**) 0, (**b**) 2, (**c**) 5, and (**d**) 10 mol% by atomic force microscopy.

To access the morphology information, atomic force microscopy was performed. All of the In-Pr-O films exhibit smooth morphologies, as depicted in Figure 4. The In-Pr-O films that were 0, 2, 5, and 10% Pr-doped had a root mean square (RMS) roughness of 0.175 ± 0.036, 0.172 ± 0.029, 0.161 ± 0.034, and 0.202 ± 0.059 nm, respectively. It is an interesting observation that atomically smooth crystalline ternary oxides could be grown from simple aqueous solution. The ultrasmooth semiconductor morphology is indispensable for TFT, since it guarantees low interface trap states as well as good ohmic contacts with the electrodes. Consequently, the near-atomic smoothness of In-Pr-O thin film shows great potential for electronic devices, as will be discussed later.

**Figure 5:**
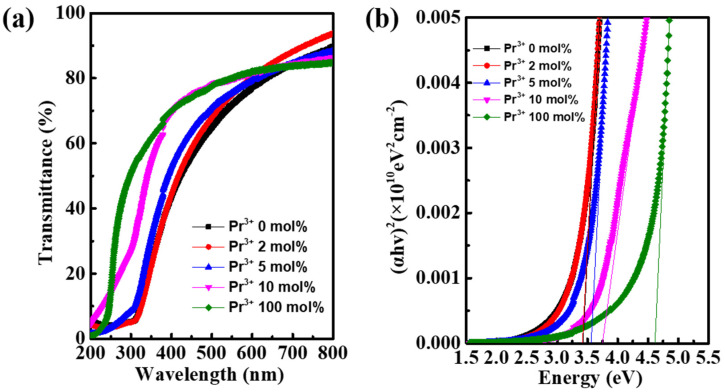
(**a**) Transmittance spectra and (**b**) evaluation of optical bandgap for In-Pr-O layer with different Pr ratios.

**Figure 6:**
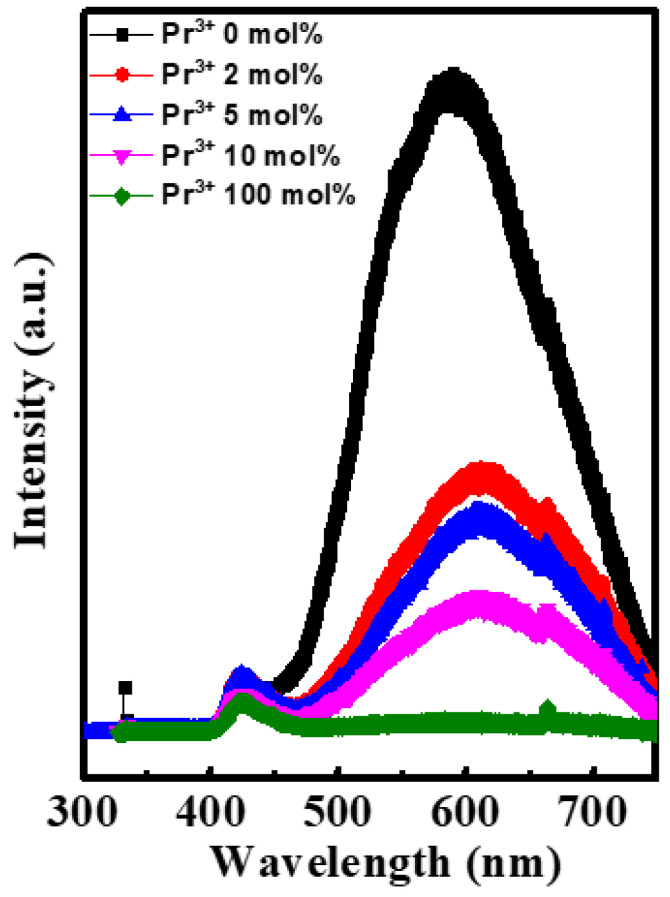
Photoluminescence spectra for In-Pr-O with indicated Pr concentrations.

Figure 5a demonstrates the optical transmittance for In-Pr-O layers with various Pr ratios. All the In-Pr-O films exhibit transmittance higher than 80%, indicating the availability for transparent electronics. Besides, the absorption edge shifts to the shorter wavelength region with the rise of Pr contents. The optical bandgap of In-Pr-O could be deduced using the Tauc formula, as illustrated in Figure 5b. As the Pr ratio increases from 0 to 10 mol%, the optical bandgap shifts from ~3.416 to ~3.747 eV. Additionally, the optical bandgap of pristine Pr_2_O_3_ was also measured to be ~4.615 eV. Therefore, the enlarged optical bandgap after Pr doping is due to the lager bandgap of Pr_2_O_3_, which would lead to the reduction of electron concentration. Figure 6 shows the photoluminescence spectra of In-Pr-O with different Pr concentrations. According to previous studies, the dominant broad peak centered at around 600 nm is ascribed to oxygen vacancy-related defects [10,18]. Consequently, the decrease of peak intensity after Pr incorporation suggests the reduction of oxygen vacancy-related defects.

**Figure 7:**
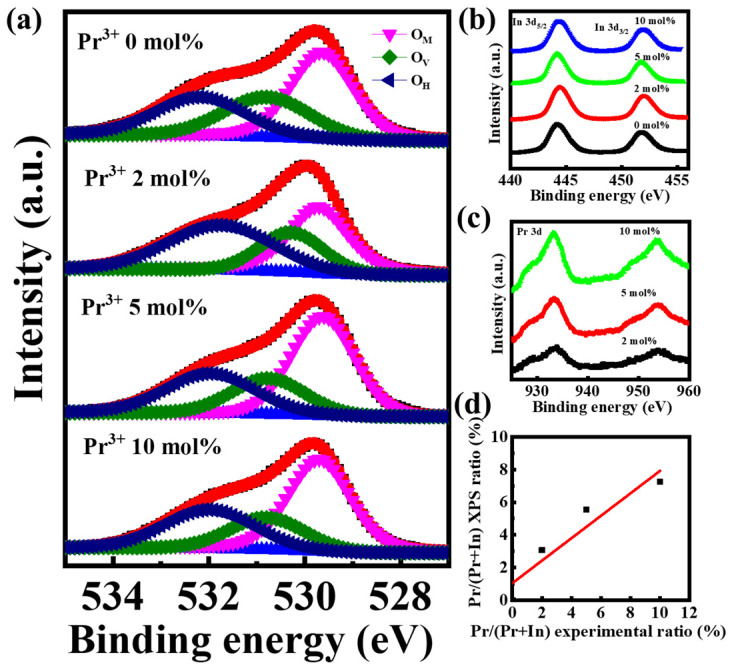
X-ray photoelectron spectroscopy spectra for In-Pr-O films with indicated Pr concentrations. (**a**) O 1s, (**b**) In 3d, (**c**) Pr 3d, and (**d**) Pr doping ratio between solution and thin film.

X-ray photoelectron spectroscopy was performed to analyze the electronic structure of In-Pr-O thin films. Figure 7a plots the O 1s peaks for In-Pr-O films with indicated Pr doping contents. The O 1s peak could be decomposed into three subpeaks centered at ~529.7 eV for the M-O-M, ~530.7 eV for the oxygen vacancies, and ~532.1 eV for the OH groups. It turns out that the ratio of oxygen vacancies decreases from ~26.16% to ~19.23% as the Pr ratio increases from 0% to 10%. This result supports the PL and UV-visible transmittance analyses’ findings that the addition of Pr could prevent the formation of oxygen vacancies and lower the carrier concentration. The standard electrode potential of Pr (−2.353 V) is lower than In (−0.34 V), the electronegativity of Pr (1.1) is lower than In (1.78), and bond strength of Pr-O (740 kJ/mol) is stronger than that of In-O (320 kJ/mol). Therefore, Pr doping could greatly reduce the oxygen vacancies in pristine In_2_O_3_. The In 3d and Pr 3d peaks are plotted in Figure 7b,c, confirming the presence of In-O and Pr-O bonding. As shown in Figure 7d, the Pr doping content in the In-Pr-O thin film is similar to that of the precursor solution.

**Figure 8:**
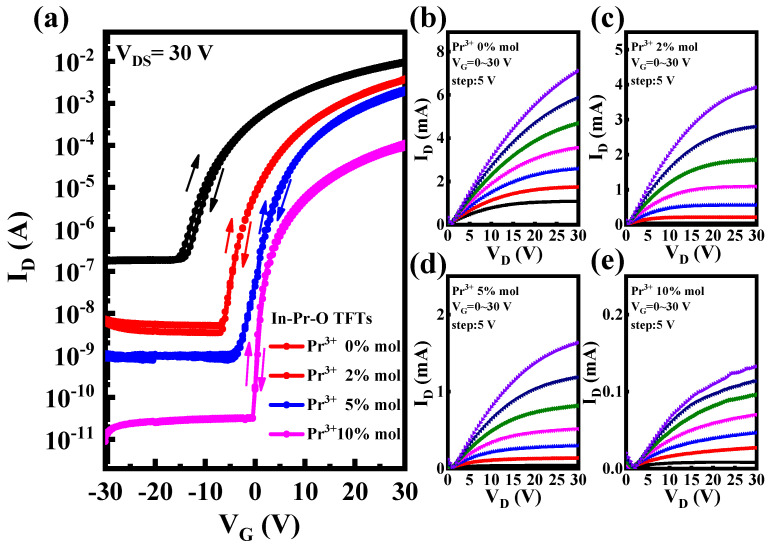
(**a**) Transfer and (**b**–**e**) output curves for In-Pr-O TFTs with 0, 2, 5, and 10 mol% Pr concentrations.

Figure 8 shows the typical transfer (V_D_ = 30 V) and output curves for Pr doped In_2_O_3_ TFTs. We measured at least 10 TFT devices for each Pr doping ratio, and obtained the corresponding statistical deviations, relevant electrical parameters were summarized in Table 1. The undoped In_2_O_3_ device has high mobility (*μ*) of 28.15 ± 1.27 cm^2^/Vs; however, it encounters high off-state current (*I*_off_) and large negative threshold voltage (*V*_T_). The *I*_off_ decreases from ~10^−7^ A to ~10^−11^ A as the Pr ratio rises from 0 mol% to 10 mol%, and the *V*_T_ shifts from −6.88 ± 0.27 V to 5.77 ± 0.48 V. The improvement of *I*_off_ and *V*_T_ is due to the reduction of carrier concentration, and originated from the suppression of oxygen vacancy-related defects. Furthermore, the subthreshold slope (*S*) also improves after Pr doping. Note that *S* value reflects the channel/dielectric interface trap states. The improved performance of *S* indicates the lowering of interface trap states also associated with oxygen vacancy-related defects. However, after Pr introduction, the In 5 s orbitals overlap decreases, resulting in the reduction of device mobility. The hysteresis of the In-Pr-O TFTs with 0, 2, 5, and 10% Pr ratios are around 1.32, 0.69, 0.44, 0.15 V, respectively. Note that as the doping amount of Pr increases, the hysteresis of In-Pr-O TFTs gradually weakens. Pr incorporation could suppress the oxygen vacancy-related defects and reduce the channel/dielectric interface trap states, leading to the improvement of hysteresis. The In-Pr-O TFTs with 5 mol% Pr demonstrate the best overall electrical characteristics, including *μ* of 17.03 ± 1.19 cm^2^/*Vs,* *I*_on_/*I*_off_ of ~10^6^, *S* of 1.32 ± 0.06 V/dec, and *V*_T_ of 4.86 ± 0.32 V, respectively.

**Figure 9:**
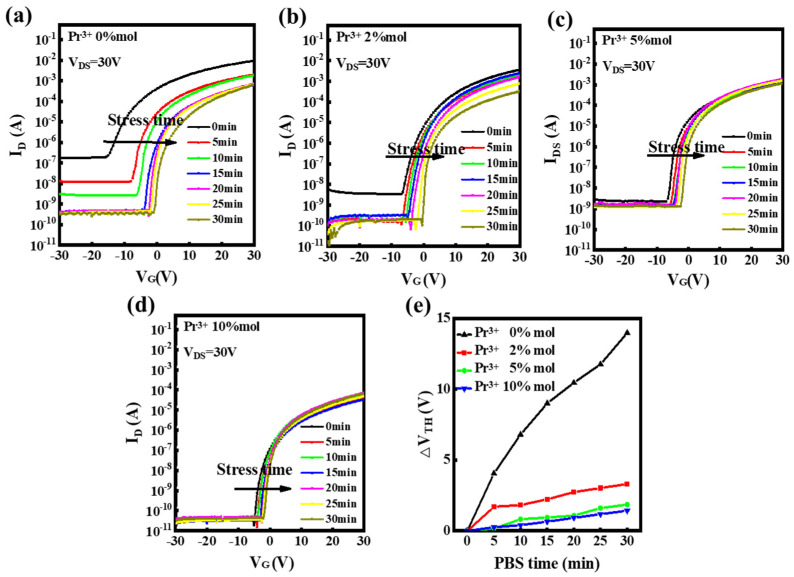
Transfer curves variations under positive bias stress for In-Pr-O TFTs with Pr mol% ratios of (**a**) 0 mol%, (**b**) 2 mol%, (**c**) 5 mol%, (**d**) 10 mol%, and (**e**) summary of *V*_T_ shifts.

Figure 9 plots the fluctuations in the transfer curves for In-Pr-O TFTs under positive bias stress (PBS, V_G_ = 20 V) for 30 min with 5 min intervals. When subjected to PBS for 30 min, the 0, 2, 5, and 10 mol% Pr-doped In_2_O_3_ TFTs show *V*_T_ shifts of around 13.96, 3.31, 1.86, and 1.43 V, respectively. According to previous research, the positive *V*_T_ shift under PBS is usually due to several factors [16,18,21]. First, the positive *V*_T_ shift is attributed to electrons trapping phenomena at the at the In-Pr-O channel or channel/dielectric interface [16,18,21]. The undoped In_2_O_3_ has high interface trap states (as reflected by the large subthreshold swing), which is related to the high oxygen vacancy-related defects. Under PBS, these defects can screen applied gate bias by capturing electron carriers from the conduction band, necessitating a greater positive gate bias in order to turn on the devices [21]. This leads to serious positive *V*_T_ shift under PBS. The Pr incorporation could suppress the oxygen vacancy-related defects and reduce the channel/dielectric interface trap states, leading to the improvement of PBS durability. Secondly, negatively charged species (O_2_^−^_(s)_) can be produced by the oxygen species absorbed from the ambient atmosphere capturing electrons in the conducting channel, expressed by O_2(g)_ + e^−^↔O_2_^−^_(s)_ [22,23], where e^−^ denotes electrons, O_2(g)_ and O_2_^−^_(s)_ represent the neutral and charged oxygen molecules in the back channel. As the electrons concentration increased under PBS, the reaction moved toward the right side of the equation [22,23]. Therefore, the positive *V*_T_ shift also arises from the consequent accumulation of O_2_^−^_(s)_ negative charges [18,22,23]. Considering that all the channel layers are extremely thin (~6 nm), the surface adsorption of O_2_ molecules may also seriously affect the PBS stability. The undoped In_2_O_3_ contains a large amount of oxygen-vacancy defects, which tend to absorb more O_2_ molecules than the Pr-doped samples, causing a larger positive *V*_T_ shift. Additionally, we note that the shift between 0 and 5 min is large under PBS especially for the pristine In_2_O_3_ TFTs, meaning there is a possibility that characteristic degradation has occurred at the beginning of the shift. Therefore, the defect creation may also occur in the undoped In_2_O_3_ sample [21]. As shown in Figure 9e, the incorporation of Pr could greatly enhance the PBS stability of the In_2_O_3_ devices, in good agreement with the hysteresis characteristics improvement shown in Figure 8 above.

**Figure 10:**
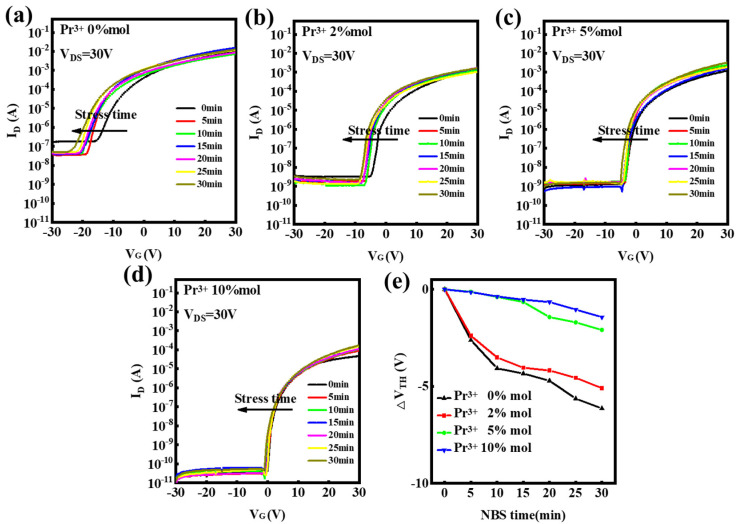
Transfer curves variations under negative bias stress for In-Pr-O TFTs with Pr mol% ratios of (**a**) 0 %, (**b**) 2 %, (**c**) 5 %, (**d**) 10 %, and (**e**) summary of *V*_T_ shifts.

Figure 10 plots the fluctuations in the transfer curves for In-Pr-O devices under negative bias stress (NBS, V_G_ = −20 V) for 30 min with 5 min intervals. When subjected to PBS for 30 min, the 0, 2, 5, and 10 mol% Pr-doped In_2_O_3_ TFTs show *V*_T_ shifts of around −6.14, −5.09, −2.09, −1.39 V, respectively. It is reported that NBS increases the number of holes in the channel layer, causing the adsorbed H_2_O_(g)_ from the atmosphere to form positively charged H_2_O^+^_(s)_ [22,23]. This is described by the following chemical reaction: H_2_O_(g)_+h^+^↔H_2_O^+^_(s)_ [22,23], where h^+^ is a hole. H_2_O_(g)_ and H_2_O^+^_(s)_ represent the neutral and positively charged water molecules in the back channel, respectively. This results in a negative *V*_T_ shift [18,22,23]. On the other hand, occupied oxygen-vacancy generates deep trap states that are widely distributed above the VBM. Under NBS, a negative gate bias will induce hole trapping at the deep level traps and a larger negative V_G_ is required for turning-on TFTs [21]. This results in the negative shift of *V*_T_. Pr incorporation could reduce the level of oxygen-related defects and hence improve the NBS stability [21].

**Figure 11:**
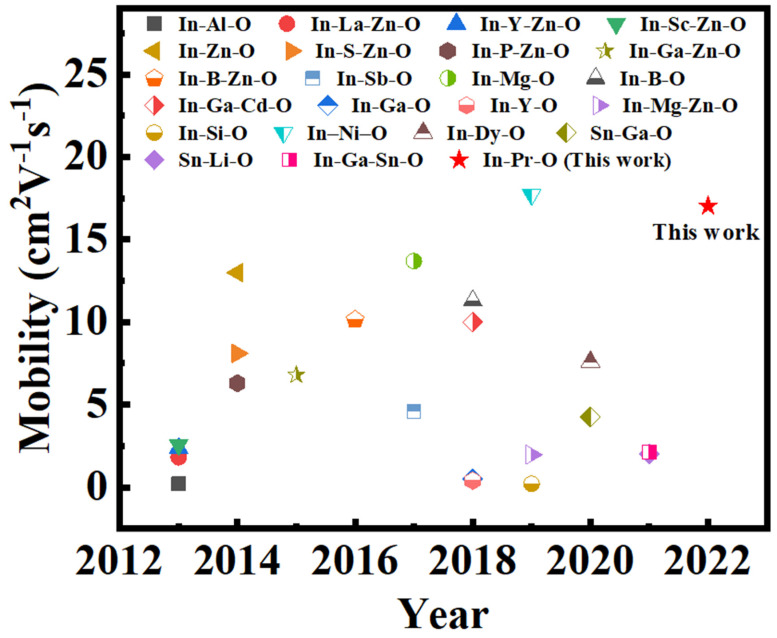
Recent advances of sol–gel oxide TFTs on Si/SiO_2_ substrate.

We summarize the recent advances of solution-derived oxide TFTs based on conventional Si/SiO_2_, as illustrated in Figure 11 and Table S1. Our novel In-Pr-O device presents state-of-the-art mobility. This achievement is due to the low electronegativity and standard electrode potential of Pr, high bond strength of Pr-O, the same bixbyite structure of Pr_2_O_3_ and In_2_O_3_, as well as the In-Pr-O channel’s atomic smooth nature. It is suggested that reducing the thickness of semiconductors to a few nanometers thick could improve short-channel immunity in scaled devices [10,24]. Therefore, our ultra-thin body In-Pr-O channel also holds great promise for next-generation scaled transistors. Moreover, reducing the thickness is known to substantially increase the flexibility of the material; thus, the In-Pr-O channel with nanoscale thicknesses could withstand high mechanical strain and thus favors flexible electronics [25].

## 4. Conclusions

We first report the synthesis and characterization of water-induced nanometer-thin (~6 nm) and near atomically smooth (~0.2 nm) crystalline In-Pr-O channel layer. Through various physical and chemical characterizations, the role of Pr doping could be summarized as follows: (1) decrease the crystallinity; (2) enlarge the bandgap of In_2_O_3_; (3) suppress the oxygen vacancy-related defects of In_2_O_3_. The incorporation of Pr has weakened the mobility of In_2_O_3_ TFT s to a certain extent. However, the other electrical parameters including on/off current ratio, subthreshold swing, threshold voltage, hysteresis, and stabilities under PBS and NBS are greatly improved after Pr doping. The optimized In-Pr-O TFT has state-of-the-art electrical performance with mobility of 17.03 ± 1.19 cm^2^/Vs and on/off current ratio of ~10^6^. The water-induced ultrathin In-Pr-O channel enables future large-scale advanced electronics with green manufacturing.

## Figures and Tables

**Table 1:** Electrical characteristics of In-Pr-O TFTs.

Pr Ratio (%)	*μ* (cm^2^V^−1^s^−1^)	*I*_on_/*I*_off_	*S* (V/dec)	*V*_T_ (V)	Hysteresis (V)	*V*_T_ Shift under PBS for 30 min (V)	*V*_T_ Shift under NBS for 30 min (V)
0	28.15 ± 1.27	5.30 × 10^4^	2.50 ± 0.13	−6.88 ± 0.27	1.32	13.96	−6.14
2	21.87 ± 1.45	7.05 × 10^5^	1.46 ± 0.09	2.37 ± 0.41	0.96	3.31	−5.09
5	17.03 ± 1.19	2.15×10^6^	1.32 ± 0.06	4.86 ± 0.32	0.44	1.86	−2.09
10	0.76 ± 0.14	1.26 × 10^7^	0.51 ± 0.11	5.77 ± 0.48	0.15	1.43	−1.39

## Supplementary Materials

The following supporting information can be downloaded at: https://www.mdpi.com/article/10.3390/nano12162880/s1, Table S1: Recent advances of solution-processed oxide TFTs based on Si/SiO_2_ substrate. The references [26,27,28,29,30,31,32,33,34,35,36,37,38,39] are cited in the supplementary materials.
