# Supplementary material for: Water-Induced Nanometer-Thin Crystalline Indium-Praseodymium Oxide Channel Layers for Thin-Film Transistors
Source: Nanomaterials (Basel). 2022 Aug 22;12(16):2880. doi: 10.3390/nano12162880 (PMC9415306; doi:10.3390/nano12162880)
Supplement: Supplementary file 1 [file nanomaterials-12-02880-s001.zip › nanomaterials-1880264-supplementary.pdf]

Supporting Information

Water-Induced Nanometer-Thin Crystalline Indium-Praseodymium Oxide Channel Layers for Thin-Film Transistors

Wangying Xu [1,*], Chuyu Xu [2], Zhibo Zhang [2], Weicheng Huang [1], Qiubao Lin [1], Shuangmu Zhuo [1], Fang Xu [3,*], Xinke Liu [2], Deliang Zhu [2] and Chun Zhao [4,*]

[1] Department of Physics, School of Science, Jimei University, Xiamen 361021, China
[2] College of Materials Science and Engineering, Shenzhen University, Shenzhen 518000, China
[3] Shenzhen Key Laboratory of Ultraintense Laser and Advanced Material Technology, Center for Advanced Material Diagnostic Technology, and College of Engineering Physics, Shenzhen Technology University, Shenzhen 518118, China
[4] Department of Electrical and Electronic Engineering, Xi'an Jiaotong-Liverpool University, Suzhou 215123, China
* Correspondence: address: wyxu@jmu.edu.cn (W.X.); xufang@sztu.edu.cn (F.X.); chun.zhao@xjtlu.edu.cn (C.Z.)

Table S1 Recent advances of solution-processed oxide TFTs based on Si/SiO_2 substrate.

Channel	Mobility ($cm^2V^{-1}s^{-1}$)	Year	Reference
In-Al-O	0.21	2013	1
In-La-Zn-O	1.81	2013	2
In-Y-Zn-O	2.37	2013	2
In-Sc-Zn-O	2.57	2013	2
In-Zn-O	13.0	2014	3
In-S-Zn-O	8.1	2014	3
In-P-Zn-O	6.3	2014	3
In-Ga-Zn-O	6.8	2015	4
In-B-Zn-O	10.15	2016	5
In-Sb-O	4.6	2017	6
In-Mg-O	13.7	2017	7
In-B-O	11.3	2018	8
In-Ga-Cd-O	10	2018	9
In-Ga-O	0.5	2018	10
In-Y-O	0.4	2018	10
In-Mg-Zn-O	1.97	2019	11
In-Si-O	0.21	2019	12
In–Ni–O	17.71	2019	13
In-Dy-O	7.60	2020	14
Sn-Ga-O	4.26	2020	15
Sn-Li-O	2	2021	16
In-Ga-Sn-O	2.13	2021	17
In–Pr–O	17.03	2022	This work

References

1. Hwang, Y. H.; Bae, B. S., Effect of Aluminum and Gallium Doping on the Performanceof Solution-Processed Indium Oxide Thin-Film Transistors. *Journal of Display Technology* **2013,** *9* (9), 704-709.
2. Hennek, J. W.; Jeremy, S.; Aiming, Y.; Myung-Gil, K.; Wei, Z.; Dravid, V. P.; Antonio, F.; Marks, T. J., Oxygen "getter" effects on microstructure and carrier transport in low temperature combustion-processed a-InXZnO (X = Ga, Sc, Y, La) transistors. *Journal of the American Chemical Society* **2013,** *135* (29), 10729-10741.
3. Park, H.; Nam, Y. Y.; Jin, J.; Bae, B. S., Improvement of bias stability of oxyanion-incorporated aqueous sol–gel processed indium zinc oxide TFTs. *Journal of Materials Chemistry C* **2014,** *2* (30), 5998.
4. Yu, X.; Smith, J.; Zhou, N.; Zeng, L.; Guo, P.; Xia, Y.; Alvarez, A.; Aghion, S.; Lin, H.; Yu, J.; Chang, R. P. H.; Bedzyk, M. J.; Ferragut, R.; Marks, T. J.; Facchetti, A., Spray-combustion synthesis: Efficient solution route to high-performance oxide transistors. *Proceedings of the National Academy of Sciences* **2015,** *112* (11), 3217-3222.
5. Zhong, D. Y.; Li, J.; Zhao, C. Y.; Huang, C. X.; Zhang, J. H.; Li, X. F.; Jiang, X. Y.; Zhang, Z. L., Enhanced Electrical Performance and Negative Bias Illumination Stability of Solution-Processed InZnO Thin-Film Transistor by Boron Addition. *IEEE Transactions on Electron Devices* **2017,** *PP* (99), 1-6.
6. Kim, T.; Jang, B.; Bae, J. H.; Park, H.; Cho, C. S.; Kwon, H. J.; Jang, J., Improvement in the Performance of Sol–Gel Processed In2O3 Thin-Film Transistor Depending on Sb Dopant Concentration. *IEEE Electron Device Lett.* **2017,** *38* (8), 1027.
7. Zhao, C. Y.; Li, J.; Zhong, D. Y.; Huang, C. X.; Zhang, J. H.; Li, X. F.; Jiang, X. Y.; Zhang, Z. L., Mg Doping to Simultaneously Improve the Electrical Performance and Stability of MgInO Thin-Film Transistors. *IEEE Trans. Electron Devices* **2017,** *64* (5), 2216.
8. Zhang, X.; Wang, B.; Huang, W.; Chen, Y.; Wang, G.; Zeng, L.; Zhu, W.; Bedzyk, M. J.; Zhang, W.; Medvedeva, J. E.; Facchetti, A.; Marks, T. J., Synergistic Boron Doping of Semiconductor and Dielectric Layers for High-Performance Metal Oxide Transistors: Interplay of Experiment and Theory. *J Am Chem Soc* **2018,** *140* (39), 12501-12510.
9. Song, A.; Javaid, K.; Yu, L.; Wu, W.; Yu, J.; Liang, L.; Zhang, H.; Lan, L.; Chang, T. C.; Cao, H., Design, Properties, and TFT Application of Solution-Processed In-Ga-Cd-O Thin Films. *physica status solidi (RRL) - Rapid Research Letters* **2018,** *12*.
10. Jaehnike, F.; Pham, D. V.; Bock, C.; Kunze, U., Role of gallium and yttrium dopants on the stability and performance of solution processed indium oxide thin-film transistors. *Journal of Materials Chemistry C* **2019,** *7* (25), 7627-7635.
11. Cheng, J.; Li, X. Y.; Guo, J.; Xu, H. F.; Chen, Y. H.; He, Y. F.; Xue, J. S.; Zhang, T.; Yu, Z. N., The role of the sequence of plasma treatment and high temperature annealing on solution-processed a-IMZO thin film transistor. *J. Alloy. Compd.* **2019,** *793*, 369-374.
12. Hoang, H.; Hori, T.; Yasuda, T.-o.; Kizu, T.; Tsukagoshi, K.; Nabatame, T.; Trinh, B.; Fujiwara, A., Erratum: Si-doping effect on solution-processed In-O thin-film transistors (2019 Mater. Res. Express 6 026410). *Materials Research Express* **2018,** *6*, 039601.
13. Li, Y.; Xu, W.; Liu, W.; Han, S.; Cao, P.; Fang, M.; Zhu, D.; Lu, Y., High-Performance Thin-Film Transistors with Aqueous Solution-Processed NiInO Channel Layer. *ACS Applied Electronic Materials* **2019,** *1* (9), 1842-1851.
14. Hong, L.; Xu, W.; Liu, W.; Han, S.; Cao, P.; Fang, M.; Zhu, D.; Lu, Y., High performance indium dysprosium oxide thin-film transistors grown from aqueous solution. *Applied Surface Science* **2020,** *504*.
15. Zhang, L.; Zhu, D.; Han, S.; Lu, Y.; Fang, M.; Liu, W.; Cao, P.; Xu, W., Aqueous solution deposition of amorphous gallium tin oxide for thin-film transistors applications. *Ceramics International* **2020,** *46* (11), 19557-19563.
16. Kim, H.-J.; Kim, D.-W.; Lee, W.-Y.; Lee, S.-H.; Bae, J.-H.; Kang, I.-M.; Jang, J., Improved Negative Bias Stress Stability of Sol–Gel-Processed Li-Doped SnO2 Thin-Film Transistors. *Electronics* **2021,** *10* (14).

17. Kim, H.; Maeng, S.; Lee, S.; Kim, J., Improved Performance and Operational Stability of Solution-Processed InGaSnO (IGTO) Thin Film Transistors by the Formation of Sn–O Complexes. *ACS Applied Electronic Materials* **2021,** *3* (3), 1199-1210.
